# The school food environment and adolescent obesity: qualitative insights from high school principals and food service personnel

**DOI:** 10.1186/1479-5868-4-18

**Published:** 2007-05-18

**Authors:** Nicole L Nollen, Christie A Befort, Patricia Snow, Christine Makosky Daley, Edward F Ellerbeck, Jasjit S Ahluwalia

**Affiliations:** 1 Department of Preventive Medicine and Public Health, University of Kansas Medical Center, 3901 Rainbow Boulevard, Kansas City, KS, USA; 2 Office of Clinical Research, University of Minnesota, 420 Delaware Street SE, Minneapolis, MN, USA

## Abstract

**Objectives:**

To examine high school personnel's perceptions of the school environment, its impact on obesity, and the potential impact of legislation regulating schools' food/beverage offerings.

**Methods:**

Semi-structured interviews were conducted with the principal (n = 8) and dietitian/food service manager (n = 7) at 8 schools (4 rural, 4 suburban) participating in a larger study examining the relationship between the school environment and adolescent health behavior patterns.

**Results:**

Principal themes included: 1) Obesity is a problem in general, but not at their school, 2) Schools have been unfairly targeted above more salient factors (e.g., community and home environment), 3) Attempts at change should start before high school, 4) Student health is one priority area among multiple competing demands; academic achievement is the top priority, 5) Legislation should be informed by educators and better incorporate the school's perspective. Food service themes included: 1) Obesity is not a problem at their school; school food service is not the cause, 2) Food offerings are based largely on the importance of preparing students for the real world by providing choice and the need to maintain high participation rates; both healthy and unhealthy options are available, 3) A la carte keeps lunch participation high and prices low but should be used as a supplement, not a replacement, to the main meal, 4) Vending provides school's additional revenue; vending is **not **part of food service and is appropriate if it does not interfere with the lunch program.

**Conclusion:**

Discrepancies exist between government/public health officials and school personnel that may inhibit collaborative efforts to address obesity through modifications to the school environment. Future policy initiatives may be enhanced by seeking the input of school personnel, providing recommendations firmly grounded in evidence-based practice, framing initiatives in terms of their potential impact on the issues of most concern to schools (e.g., academic achievement, finances/revenue), and minimizing barriers by providing schools adequate resources to carry out and evaluate the effectiveness of their efforts.

## Background

The prevalence of overweight among US children and adolescents (6–19 years of age) has reached epidemic proportions [1]. Approximately 16.5% are overweight (body mass index (BMI) ≥ the 95th percentile for age and gender) and 31.5% are considered at risk for overweight (BMI ≥ 85th but < 95th percentile). Escalating obesity rates threaten the health of our nation and the social, emotional, and physical well-being of our youth. Poor diet and physical inactivity account for approximately 365,000 deaths per year, ranking second only to tobacco as the leading cause of preventable death [2] and cost our nation an estimated $110 to $129 billion annually in direct and indirect health-care costs [3]. Overweight youth are more likely to become obese adults [4] and to experience significant obesity-related health concerns even as normal weight adults [5]. Overweight children are also more likely than their normal weight peers to experience lower self-esteem, depressed mood, body dissatisfaction, discrimination, negative stereotyping, and social marginalization [6].

Slowing the prevalence of childhood and adolescent obesity has become a national priority. Given that more than 95% of American youth attend school and consume as much as 35%–40% of their daily caloric intake there [7], schools have been regarded as an ideal setting for prevention and treatment efforts. A growing body of literature has begun to provide a snapshot of the school food environment and its relationship to youth dietary practices and obesity. Findings suggest that 90% of schools offer an à la carte (ALC) lunch program [7], and over 80% of high school students have access to vending machines, school stores, snack bars, or canteens [8], which offer items consistently found to be low in nutrients and high in fat, calories, and sugar [8,9]. Although schools also offer healthy items (e.g., fruits and vegetables, low fat snacks, 100% fruit juice, low fat milk, bottled water), studies show they are less likely to be available and less likely to be purchased than other foods and beverages [10,11]. Evidence also suggests a link between school policies, dietary habits, and obesity. Specifically, in a recent study of high school students, those at schools permitting open campus lunch were significantly more likely to eat lunch at a fast food restaurant than students at schools with a closed campus lunch policy [12]. Additionally, student food/beverage purchases were significantly related to the number of vending machines permitted, their hours of operation, and the types of food/beverage allowed in the machines. Although preliminary, a separate study found a positive relationship between the BMI of middle school students and the number of food practices permitted at their school (e.g., use of food as an incentive or classroom reward, classroom fundraising) [13], indicating a possible relationship between the school environment and adolescent obesity.

Given the current state of the school environment and its possible relationship to youth dietary practices and obesity, many national, state, and local initiatives are underway to create healthier school environments [14]. In 2004, the federal government passed the Child Nutrition and Women, Infant, and Children (WIC) Reauthorization Act requiring all schools with a federally funded meals program to develop and implement a wellness policy for the 2006–2007 school year [15]. At a minimum, policies must include nutrition guidelines for foods available on campus during the school day, provide assurance that school meals meet US Secretary of Agriculture Guidelines, and establish a plan for measuring implementation of the policy. Many states, including Kansas, passed additional legislation recommending specific nutritional guidelines for schools to use in developing their wellness policy [14]. In addition, 12 states are considering BMI reporting legislation and 5 are currently monitoring student BMI. Other policy initiatives, including restricting access to and sales of competitive food and beverage items, particularly soft drinks, increasing and promoting access to fresh produce in schools, and establishing school wellness committees, are being considered or are underway in 28 states [14].

While evidence suggests schools may be a contributing factor to the obesity epidemic [10-13] and legislation to create healthier school environments is underway [14,15], no known studies have examined the perspective of school personnel. Understanding their perspective is critical to progress being made toward collaborative efforts addressing adolescent obesity. Therefore, the purpose of this qualitative study was to examine high school personnel's perceptions of the school environment, its impact on obesity, and the potential impact of legislation regulating schools' food/beverage offerings.

## Method

### School recruitment

Schools were recruited from a larger, observational and survey study examining the relationship between the school environment and student dietary, physical activity, and weight patterns. To be eligible for the qualitative study, schools were required to have at least one vending machine and an ALC lunch program that offered items (e.g., pizza, chips, cookies, beverages) separate from those sold on the reimbursable lunch program. For the purposes of this paper, reimbursable lunch was defined as lunch offered through the US Department of Agriculture National School Lunch Program (USDA NSLP). Meals offered through this program must meet federally mandated nutrition guidelines, which include ≤ 30% of calories from fat and < 10% from saturated fat and providing one-third of the Recommended Dietary Allowances of protein, Vitamins A and C, iron, calcium, and calories [16]. A la carte and vending were defined as food and beverage items sold separate from the USDA NSLP [17]. Vending included those food and beverage items sold through a machine. A la carte included all other competitive food and beverage items sold in the cafeteria and/or at snack bars.

Of the 19 high schools participating in the parent study, 8 met the eligibility criteria and were invited to participate. Schools were excluded because they did not offer ALC lunch (n = 7) or had a limited ALC lunch program (e.g., offered only milk or extra portions from the reimbursable lunch line; n = 3). In addition, 1 private school was excluded because their food service structure was not representative of the larger sample or public school food service, in general. All eight schools (4 rural, 4 suburban) invited to participate agreed. Rural/suburban status was determined by the National Center for Education Statistics locale codes of participating schools.

### Procedure

Semi-structured interviews were conducted separately with the principal (n = 8) and dietitian/food service manager (n = 7) at each of the 8 participating schools. Among the food service interviews, 3 were conducted with the school's registered dietitian (one dietitian served 2 of the participating schools). The remaining 4 schools, all rural, did not have a dietitian; therefore interviews were conducted with the food service manager. Two clinical psychologists and one public health professional, all with previous training and experience in qualitative interviewing, conducted the interviews. Interviews followed a semi-structured format whereby a standard guide of open-ended questions was used to stimulate thought and discussion about the school food environment and its relationship to student health. The guide was developed according to published methodological suggestions by researchers with experience working with schools and in semi-structured interview methodology [18]. Table 1 displays primary questions from the interview guide. Interviews took approximately 1 hour to complete.

**Table 1:** Moderators Guide: Overview of Major Topics and Questions

*Principal Interviews*	
**Topic**	**Questions**

School's Focus	• Schools have many areas of focus. What is the importance placed on each area at your school? Why?

Student Health	• What role, if any, do you feel school's play in the health of students? • How, if at all, do you think your schools food practices influence the health of students? • What one thing is most important to change in your school to promote the health of your students? Why?

Obesity	• Do you consider overweight/obesity to be a problem among students at this school? Why/why not? • What, if anything, can schools do to help reduce the rates of childhood/adolescent obesity? • How do feel about the media's portrayal of schools' food environment and its role in childhood/adolescent obesity?

Government mandates	• How do you feel about the state's push for schools to establish a wellness policy? What impact, if any, will this have on your school?

*Food Service Personnel Interviews*	

**Topic**	**Questions**

Food Service Focus and Food Offerings*	• What goes into the decision about what items to serve? • How do you balance the role of providing options and encouraging students to make healthy choices with providing access only to healthy foods? • What changes, if any, have you tried/would like to try to encourage healthier dietary habits among students?

Obesity	• Do you consider overweight/obesity to be a problem among students at this school? Why/why not? • What role, if any, do you feel the school food environment plays in childhood/adolescent obesity? • How do feel about the media's portrayal of schools' food environments and its role in childhood/adolescent obesity?

Government Mandates	• How do you feel about the state's push for schools to establish a wellness policy? What impact, if any, will this have on your school's food service program?

Challenges/Barriers	• What challenges, if any, do you encounter on regular basis that make your job difficult? • In an ideal world, what would the school food service program at this school look like? What would your role be?

*NOTE: Food offering questions were asked separately for the reimbursable lunch, a la carte lunch, and vending programs

Prior to data collection, all participants provided informed consent and permission to be audio-taped. Participants received a $40 Target gift card as compensation for their time and effort. All study methods and protocols were approved by the University of Kansas Medical Center's Human Subjects Committee prior to implementation.

### Data analysis

Audio-recordings of the interviews were transcribed verbatim by a contracted professional transcription service. All transcripts were checked by interviewers for completeness and accuracy prior to data analysis. A medical anthropologist with over 10 years of qualitative experience led the analysis and trained three independent coders. Coders first deductively categorized verbatim transcripts by hand into major topic areas using initial codes developed by the research team based on the interview guide. Six major topic areas were identified for the principal interviews and five for the dietitian/food service manager interviews. Coders then inductively or "open" coded by hand within each major topic area using a grounded theory approach whereby categories and concepts emerge from the text and are then linked together [18]. This approach allows the data to speak for themselves, generating new theories and providing insights that are not possible when using an established theory a priori. A fourth independent researcher cross-checked inductive codes, identified minor discrepancies and different terminology used by each coder to describe the same content, and identified major themes within codes. Cross-checking provides a measure of how well the data are indexed and, thus, gives a qualitative measure of inter-coder reliability [19]. Overall, the independent researcher found high inter-coder reliability. The research team then met as a group to discuss the major themes and reach consensus; all major saturated themes were identical across coders.

## Results

Student demographic and school environmental characteristics of participating schools are displayed in Table 2. Schools participating in the parent study, but excluded from the present analyses (n = 11), were smaller [238 students (SD = 170) versus 600 students (SD = 305), p = 0.004], had more students eligible for free or reduced lunch [29.6% (SD = 14.9) versus 13.0% (SD = 5.5), p = 0.008], and had a larger percentage of students with BMI ≥ 85^th ^percentile (33.7% versus 22.7%, p = 0.02).

**Table 2:** Student Demographic and School Environmental Characteristics

		School Type^a^	
	Total (n = 8 schools)	Suburban (n = 4 schools)	Rural (n = 4 schools)

*Student Demographics*			
Enrollment, mean (SD)	600 (305)	661 (188)	539 (415)
Race, % White students	87.4%	87.6%	87.2%
Gender, % male	49.7%	49.1%	49.6%
Free/reduced lunch status, %	12.6%	10.5%	15.2%
% students ≥ 85th BMI percentile^b^	22.7%	17.4%	25.3%
*School Environmental Characteristics*			
Vending machines available for student use, mean (SD)	5.9 (2.6)	6.8 (2.8)	5.0 (2.5)
ALC products offered, mean (SD)	56.0 (36.9)	76.3 (42.9)	35.8 (15.5)

^a^Rural/Suburban status was determined using the National Center for Education Statistic's designated locale codes

^b^Derived from a randomly selected sub-sample of 110 students at the participating schools

### Thematic analysis

#### Themes derived from principals

Across principal interviews, 5 themes encompassing 3 primary topic areas emerged (see Table 3).

**Table 3:** Semi-Structured Interview Topics and Major Themes

*Principal Interviews*	
**Topic**	**Themes**

Obesity	1. Obesity is a problem in general, but not at their school. 2. Schools have been unfairly targeted above more salient factors such as community and home environments. 3. Dietary habits are largely established by the time students get to high school; attempts at change should start earlier.

School Priorities	4. Schools have a role in student health, although academic achievement is the top priority among many competing demands.

Government Legislation/Mandates	5. Additional legislation (i.e., the establishment of local wellness policies) could be helpful, although mandates should be informed by educators and better incorporate the school's perspective.

*Food Service Interviews*	

**Topic**	**Themes**

Obesity	1. Obesity may be a problem in general, but not at their school. 2. School food service is not the cause.

Food/Beverage Offerings	3. The reasons for food offerings are multi-factorial and are based largely on the perceived importance of preparing students for the real world by providing choice and the need to maintain high participation rates; both healthy and unhealthy options are available.

ALC and Vending	4. ALC is valuable; it keeps lunch participation high and prices low but should be used as a supplement, not a replacement, to the main meal. 5. Vending is also valuable in that it provides school's additional revenue; vending is not part of food service and is appropriate if it does not interfere with the lunch program.

##### Obesity

Principals agreed that obesity is a problem among high school students, in general; however most did not feel that obesity was a problem at their school. Several cited instances of students who were overweight or obese but felt they were exceptions and not the rule. As a whole, principals felt their student body was fit and active, citing that a large percentage were involved in athletics, cheerleading, drill team, elective PE, and/or extracurricular activities. For many, obesity was not seen as a problem until it interfered with students' academic abilities. For example, one principal noted, "As far as a problem, I have not really seen a big problem. We don't seem to see the difficulty for a kid if they are overweight, probably because of the tasks we ask students to do, which are mostly academic."

Principals also agreed that schools have an influence and that they should be part of the solution. They did not, however, feel that adolescent obesity was the fault of the school. As one principal noted, "I think we have a significant influence but I don't think we could be the cause or the cure." Principals believed that schools had been unfairly targeted, particularly by the media, and that other pervasive social factors, namely community, home environments, and society, are greater influences that have been largely overlooked. As put by one principal, "Kids are desensitized to good eating and good health habits...because we live in a drive up, drive by, and drive through society." Another noted, "The school should be a part of what's going on, I just don't think we should be looked at as the savior to any particular social issue... I think we need to be a part of it, but don't look to us to solve all of those problems. I think we act as parents way too much." Principals wanted schools to be *part *of the solution, in collaboration with parents and they community; they did not want schools to be the *only *solution.

Finally, principals felt dietary habits were largely established by the time students got to high school and that attempts at change should be started earlier. One noted, "It's kind of like a reading program. It's nice to say we're stressing reading at the high school level but that's too late...The same goes for lifestyle...If they're allowed to sit at a computer or video game for three or four hours a day, I guarantee you they are not going to change some of those things when they get older..."

##### School priorities

Principals felt student health was important but most did not consider it the school's top priority. In particular, they cited multiple competing demands (e.g., academics, discipline, safety/learning environment, athletics/extracurricular activities, physical and mental health) and discussed the difficulty in striking a balance between the many roles and functions served by the school. Among these multiple demands, principals considered academic achievement their top priority. Physical and mental health was also ranked highly, primarily because of the connection principals drew between good physical/mental health and students performing their best academically. As stated by one principal, "The approach we've always tried to take is when it (mental and physical health) becomes an issue and effects the other three (learning environment, academic environment, discipline) we get involved...they've all got to fit together...I don't think you can zero in on any one without looking at the big picture."

##### Government legislation/mandates

Principals were ambivalent about state mandates requiring them to establish a school wellness policy and set limits on the foods/beverages offered at their school. However, many felt the mandates could be helpful if instituted correctly. One principal stated, "I think any standard that people feel would be in the best interest of teenagers...I would be for it. If they feel there is something that would help students with their health and fitness and be able to control obesity, I would certainly be in favor of it." On the other hand, they felt burdened by the many demands placed on them by the government and believed that legislation was largely uninformed by educators and did not incorporate the school's perspective. Reflecting this sentiment, one principal noted, "I think it's a good thing but I think they need to make sure recommendations are taken from educators...that's the thing that probably disturbs me more than anything about the legislature is that they mandate things when they really haven't talked to people in the field..."

Principals also talked about the difficulties created by unfunded mandates. Specifically, most believed the establishment of a school wellness committee would place additional demands on staff and teachers' time. They also believed it would be difficult to institute a comprehensive and effective wellness plan (e.g., more time devoted to nutrition education, changes to the food/beverages offered) without the proper resources. For example, one principal said, "I'm torn...I'm mandated to do more instruction, I'm mandated to move all of these kids...and I'm just getting another mandate that I can't foot the bill on. To do it right, I need more resources...You can't throw me a wellness program and say, but there's no money, and that's what we continually see from the government." Overall, principals felt the addition of another mandate/demand meant that something else would have to be given up, as their school could not continue to add programs or curricula without other responsibilities being taken away. This was summed up by one principal who said, "It is important for us to have healthy kids...but what are we willing to give up to get it?"

#### Themes derived from dietitians/food service managers

Across dietitian/food service manager interviews, 5 themes encompassing 4 primary topic areas emerged (see Table 3).

##### Obesity

Food service personnel believed that obesity may be problem among high school students, in general; they did not feel it was common at their school. They also felt strongly that the school food environment was not the cause and, instead, attributed the rise in adolescent obesity to external factors including the home environment and the 'culture' of physical inactivity. As noted by one dietitian, "I don't see schools as contributors to childhood obesity. We can't... we're 1/3 of what the child eats in a day for 170-some days a year...We don't have that much input into it." Reflecting a similar sentiment, others noted that students were becoming overweight as children and that the 'American lifestyle' and the home environment were the early causes of obesity, not the school environment.

Food service personnel believed they were doing the best they could to provide a healthy meal for students. Although they viewed the school food environment as part of the solution for overweight/obesity, they felt strongly that it should be a shared responsibility. As noted by one dietitian, "They've put a lot of responsibility on the schools to take care of issues in the school environment and I think that, you know, it should be a shared responsibility. I think homes should share in this responsibility with the school...I don't think it should be pushed as a school problem. I think it needs to be a community addressed issue." Another stated, "We're doing everything we can to educate that child in every way that we can...I see myself as a piece in the larger education puzzle, but it's the parent that puts the puzzle together." In general, personnel wanted more involvement with parents.

##### Food/beverage offerings

Food service personnel noted the reasons for food/beverage offerings were multi-factorial; based largely on the perceived importance of preparing students for the real world by providing choice and the need to maintain high participation rates. Specifically, food service personnel believed that one of their roles was to prepare students for the 'real world' by educating students, offering a wide variety of selections, and then, ultimately, allowing them to make choices. Some stressed that this was their role at the *high school *level; in elementary and middle school they believed their role was less about choice and more about education and ensuring healthy eating. Personnel noted offering an approximately equal proportion of healthy (e.g., fresh fruits and vegetables, baked chips, low fat milk, frozen yogurt, 100% fruit juice) and unhealthy (e.g., regular chips, cookies, candy bars, pizza, hamburgers) items through ALC and vending and believed that limiting choice would interfere with their role. As noted by one foodservice manager, "Make it accessible to them so they can make those choices. I mean they're going to have to make choices all their life...I think it will make them better. They'll just be more ready when they get out there to make those better choices." Another said, "...If you've got a candy bar sitting next to an apple, that kid reaches for the candy bar. That's just today's society and that's part of my job is to train them...I mean it's all part of the education process is teaching life skills...and hope that someday they'll say, 'Oh, I'd rather have the apple'."

Personnel noted that the items offered were based largely on student preference, as well as the need to maintain high participation rates and minimize cost and waste/spoilage. Specifically, they mentioned trying to maintain a certain participation level for reimbursable lunch and stated that if items were offered that students didn't' like, they wouldn't eat. Personnel also noted that if students didn't like the lunch menu they would bring items from home or get food elsewhere (e.g., a convenience store). All felt the chances of a child eating healthy were greater if meals were purchased at their school. As noted by one dietitian, "We try to maintain a certain participation level...and we try to stay at, at least 70% participation in the school meal program. If my participation drops, I know immediately...If students don't like the menu they bring a sack lunch and it reflects in my numbers and I go, oh, maybe we ought to revamp the menu." Another stated, "As far as the reimbursable meals it has to do with what I think the kids will eat, because if they don't eat they're not getting the nutrition anyway. I can serve it, and I'll meet my requirements, but if they're not eating it then we're not fulfilling our mission of having a healthy child. So I pick things that I think the kids will eat."

Personnel discussed the importance of making an equal portion of healthy and unhealthy items available to facilitate choice, but also noted their struggles with this approach. Food service personnel largely believed they offered 50% healthy and 50% unhealthy items. They spoke about barriers to offering a greater percentage of healthier items. In particular, the issues of cost and minimizing waste/spoilage were salient. As stated by one food service manager, "If she (the head cook) makes it and it doesn't sell, that could be $100 worth of stuff just wasted everyday...We have done fruit...well it just sits out there. You know, bananas and apples, the kids asked for them, we tried them for two weeks...the bananas, of course, didn't last long and we threw them out." Another described her decision to continue to offer fresh fruits and vegetables as a gamble. Although she stressed the importance of offering them, she noted, "When you have packaged potato chips you have no loss as long as it doesn't go out of date. But...a fresh apple, you could have loss, and that's a gamble." Personnel also acknowledged that unhealthy items are chosen more frequently than the healthier alternatives. As mentioned by one dietitian, "You know what they're going to take. We put out 12 apples and we'll get 11 of them back. We put out 200 cookies and they'll all be gone." When describing reimbursable lunch, a food service manager said, "They have to take three things of the five and a vegetable usually isn't their choice."

Finally, many mentioned wanting food service to be liked and appreciated by the students. As stated by one food service manager, "I want them to miss us when they're gone. There are a lot of them that are like, wow...I griped about it when I was there but I really miss certain things." They described students as their customers and their job to satisfy student requests, within reason. Within this context, the importance of open, honest feedback from students was discussed. Many mentioned regularly seeking and implementing student's input, particularly when new items were offered or when items were offered in a different way (e.g., canned fruit versus fresh fruit versus fresh fruit prepared as a fruit cup).

##### ALC and vending

ALC was seen as valuable, primarily because it helped to keep lunch participation high and reimbursable meal prices low. Some described it as essential to the revenue of the food service program. For example, one dietitian stated, "If you really get down to the crux of the matter, ALC generally supports the school lunch program. For instance, we charge $1.75 for lunch...I get reimbursement for that at $.44 and, you know, I can't truthfully fix lunch for that amount of money." Another stated, "Our ALC plays a big part in the finances of our district...if we didn't have the ALC there would be a big change in where all the money comes from to do the school lunch." Others noted that ALC was more about providing something for students to eat if they did not like the reimbursable menu. They stressed that many of their kids were active in before and after school activities and that it was critical for students to eat something for lunch. One food service manager stated, "I think eating is better than starving all day long...they can order the lunch. If they don't like the lunch, they can order a salad. If they don't like either one, they know there is a la carte to have something to eat that day." Nearly all personnel stressed that ALC was intended as a *supplement*, and not a *replacement *to the main meal. As stated by one dietitian, "The ALC program, truthfully, it is not supposed to be used as an entire meal...The idea is to get a lunch and then if you want a cookie or chips or something to go with it, then that's the appropriate use of ALC." Consistent with this philosophy, many snack-type items were provided (e.g., chips, beverages, and desserts). Some schools also opened ALC only after students had gone through the reimbursable line.

Vending was also seen as valuable for many of the same reasons. Food service personnel pointed out, however, that vending was not part of their food service program and they had no input into the vending items offered. Specifically, personnel noted that vending was under the control of the administration or student groups. They were fine with not having input into vending; some personnel noted that they did not want the negative attention from it either. As stated by one dietitian, "The purpose of them (vending machines) primarily is a source of funding...and it does make my department look to somebody from the outside...If people know that that part of it is not mine then I don't mind. If I don't get the revenue from it, I don't want the bad press from it either." Personnel believed vending was appropriate as long as it did not interfere with the food service program. One dietitian noted an instance where a meal replacement shake was removed from vending because of her concern that students would buy the shake in place of the school lunch. Regarding this instance she stated, "So input on the vending machine...I don't want anything competing with my school lunch...that's as far as I feel my input should go at this point."

#### Unsaturated themes derived from dietitians/food service managers

An additional 2 unsaturated themes (i.e., not expressed by all personnel) emerged from the food service interviews (not displayed in Table 3).

##### Government legislation/mandates

There was little consensus regarding the potential impact of state mandates on their food service program. Some felt the quality of the food/beverage items they offered met or exceeded nutritional standards and, therefore, believed the mandates would have little impact. Others questioned the reasoning behind the mandates or felt their impact could be detrimental. As stated by one dietitian, "I think they would find that the vending and ALC are not the problem. I mean if you're trying to fix childhood obesity...it's not the vending, it's not the ALC, it's the lifestyle." Another expressed concern that limiting competitive food items would differentially impact low income families. She stated, "I understand, you know the nutritional aspect...behind eliminating all of that. I think for districts like mine where we have a low free and reduced lunch population that students would end up bringing more stuff from home and the lunch program would end up being more of a program that just free and reduced lunch kids participate in."

##### Satisfaction

Many felt they were doing the best they could with limited resources. Several believed that improvements could be made. One dietitian acknowledged, "I guess I have a hard time thinking that the ALC choices are actually healthy, they're just...we try and restrict how unhealthy they are." Many positive changes had been made in some of the schools. For example, one school encouraged kids to try healthier alternatives of popular items (e.g., baked versus regular chips; fat free frozen yogurt versus ice cream; skim milk versus whole milk) by offering free taste tests or including these items as part of a meal deal. Another charged less for healthy items (e.g., $0.50 for baked chips; $0.75 for regular) and had set nutritional standards for all of the items offered through ALC. Another opted to bake most items from scratch, which allowed more control over the nutritional content and portion size. The food service in this school had also begun taking an active role in teaching portions of the nutrition education curriculum, as well as having monthly 'health nights' to facilitate the interest and involvement of parents.

## Discussion

Our findings suggest a number of consistent themes among high school principals and food service personnel regarding the school environment and its impact on obesity. Both principals and food service personnel believed that obesity was a problem in general; however, they did not feel it was a problem at their school, despite nearly one-fourth of students having a BMI ≥ 85th percentile. These findings mirror data from the family literature, which suggest that parents struggle to accurately identify their child as at risk for or overweight [20-22], and indicate a need to increase obesity awareness among school personnel and parents. Despite concerns about privacy, stigmatization, and appropriate follow-up services, the Institute of Medicine endorses BMI reporting by schools [23] and data support the effectiveness of this practice. Specifically, in a recent study, parents of overweight children who received a health report card of their child's BMI percentile and risk category were more aware of their child's weight status and health risk and were more likely to consider seeking appropriate dietary, physical activity, and medical care compared to parents not receiving this information [24]. Many schools already complete annual height, weight, or body mass screenings [25]. Generating BMI percentiles and notifying parents of the results represents a logical next step towards increased awareness and improved surveillance and early prevention efforts involving both parents and schools.

All personnel felt schools had been unfairly targeted as the cause of adolescent obesity, and they believed that schools should be only *part *of a larger solution that included parents, government, and communities. It is important to note the many principals expressed discomfort describing obesity as a 'problem,' citing that it was a 'problem' only when it began to interfere with students' physical and mental health and/or their academic achievement. This distinction is important and represents a possible disconnect between the perceptions of public health and government officials and school administrators. The majority of school-based research and/or policy-based initiatives are currently framed in terms of obesity prevention and wellness; administrators may be more likely to engage in these initiatives if they are seen as positively impacting the factors of most importance to their school, including student's physical and mental health and/or their academic achievement.

Although both principals and food service personnel believed schools should be a part of the solution, they offered different perspectives regarding the specific roles schools could play. Principals largely focused on changes to the overall structure of the school day, which included adding more structured wellness and/or nutrition/physical activity instruction. Food service personnel focused heavily on education as a way to address obesity and the importance of choice in the education process. The food/beverage offerings seemed largely governed by their mission of educating through choice. While some acknowledged that improvements to the food environment could be made, the majority expressed satisfaction with their food service program and saw little need or room for change. Almost universally, food service personnel mentioned that at least half of their ALC food items were 'healthy,' although definitions of what constituted a 'healthy' item differed. Most also mentioned that students choosing to eat healthy could certainly do so. Interestingly, many personnel believed that limiting choice would interfere with the education process, and although not explicitly stated, was perceived by some to be a disservice to students.

The perceived role of educating through choice is in contrast to many of the current policy-based initiatives, which seek to limit choices, and to much of the literature on the impact of availability on students' choices and dietary intake. Within the school environment, the availability of energy dense, nutrient poor foods through ALC and vending has been found to adversely impact the nutritional quality of students' diets by displacing the consumption of healthy foods (e.g., fruits, vegetables, milk) and contributing to excess fat intake [11,26,27]. Additionally, school wide practices supporting the consumption of high calorie, low nutrient foods have been positively associated with student BMI [13]. Before modifications to the school food environment are successfully implemented, it may first be necessary to educate food service personnel on the relationship between competitive food availability and the nutritional quality of student's food choices. Additionally, schools must feel confident that providing healthful food choices will not adversely impact revenue, lunch participation rates, or school meal costs. Unfortunately, research in this area is limited and more work is needed before collaborative efforts addressing adolescent obesity through modifications to the school food environment can be successfully undertaken. Additional training regarding nutritional standards and what constitutes a 'healthy' item may be warranted. Education standards do not currently exist for food service personnel and, as a result, knowledge about nutrition and its related concepts varies widely [28]. Establishing education standards seems critical to increasing foodservice personnel's awareness of the nutritional quality and health issues associated with the types and quantities of foods they serve and may go a long way toward ensuring a healthier school environment [28,29].

Similar to the food service personnel, some principals felt that mandates for school wellness policies *could be *helpful, but most believed this was not the best approach to addressing adolescent obesity and would be largely ineffective without the proper assistance and resources. As a result, many principals viewed the wellness policy as a hassle and felt that future policies focused on the school environment and adolescent obesity would be more effective if they: a) were informed by educators who were experienced 'in the field;' b) provided adequate time and resources for schools to develop and carry out changes; c) lessened schools' responsibilities in other areas (e.g., academic achievement) and; d) reached beyond the school environment to address the foods/beverages available to students at restaurants and convenience stores located within close proximity to schools. Principal's suggestions are consistent with a recent statement released by the Society of Behavioral Medicine, which suggests that, although the Child Nutrition and WIC Reauthorization Act of 2004 is a positive step, future legislation must encourage schools to better incorporate evidence-based methods for promoting behavior change and provide schools with funding to adequately carry out and evaluate the effectiveness of initiatives [30].

Obtaining buy-in and removing barriers among principals and food service personnel are critical steps in efforts to change the school food environment. Relevant barriers, discussed by our sample and consistently mentioned as barriers in the literature, included the increased cost, perishable nature, and difficulty in obtaining healthy items, the possibility for decreased lunchtime participation and its potential impact on rising meal costs, the importance of ALC in supporting the food service program, low priority for health promotion activities due to time constraints and competing demands, and budgetary constraints that compel schools to find additional funding to support necessary programs [31,32]. While several studies have demonstrated that pricing strategies increase the sale of healthy items (e.g., fresh fruit, baby carrots, low fat snacks) [33-35] and do not adversely impact revenue [36], the methods of these studies are difficult to translate or sustain in 'real world' settings. To obtain buy-in and support from school personnel, more practical strategies for addressing relevant barriers are needed. Strategies might include providing financial incentives for offering healthier items, working with vendors to make healthy items readily available at a lower cost, decreasing reliance on ALC by increasing federal reimbursement for the main lunch program, and educating personnel about how to make healthy, yet palatable changes to their existing offerings.

Personnel's reaction to legislative involvement in the school environment mirrors recent debate ignited by obesity-related public health laws. While advocates view obesity-related policy initiatives as a powerful instrument of public health, opponents see these measures as an infringement on freedom of choice [37]. The perspective of the school personnel in this study represents yet another disconnect between government and public health officials and educators. Personnel had heard about the mandate but knew very little about the specific details of what it entailed. This raises concern about how policy level changes are communicated to school personnel and the degree to which legislation is taken seriously. Many seemed to disregard the legislation as 'yet another mandate being handed down by the government' or 'yet another thing schools are being told we have to do.' Others seemed to resent the authority imposed on them by federal and state government and expressed a desire for more input into the process. Given these reactions, the effectiveness of the mandate in creating a healthier school environment is called into question. As additional legislation is considered and proposed, it seems necessary to better engage school officials and equip them with the proper resources to successfully carry out proposed changes [30].

Two final points emerged that are worth noting. Almost universally, food service personnel stressed that ALC was intended as a supplement, not a replacement to the main meal. The programs in many schools were set up in this fashion (e.g., offered only snack-type items, opened only after students had gone through the main lunch line); however it is not known if this is how it was being used. More research is needed to establish student's ALC lunch patterns. In addition, there are some inherent problems with ALC being used as a supplement to the main meal. Specifically, previous research has shown that the items available from ALC are low in nutrients and high in fat, calories, and sugar [8,9] and the availability of these items has been found to displace the selection of other healthful foods and adversely impact the quality of student's diets [11,26,27]. These findings are concerning and suggest that ALC items, regardless of whether consumed as a meal or as a supplement to a meal, provide excess calories and fat and may pose a threat to the overall healthfulness of student's diets. More research examining these associations is critical, as is the communication of the findings to school officials.

Finally, food service personnel stressed that vending was not a part of the food service program and, in general, they wanted little input into vending unless it interfered with main lunch or ALC purchases. Principals also seemed largely removed from the vending programs, raising questions about who provided oversight for vending in the majority of participating schools. In many cases, it appeared that decisions regarding what products to sell were left primarily to the vendors. Given that changes to the products available in school vending machines are a relatively simple, yet potentially fruitful way to facilitate healthier school environments, more effort should be devoted to identifying the appropriate personnel within schools and encouraging them to take a more active role in product selection for their vending programs.

A number of study limitations should be noted. First, the results provide an in-depth understanding of a relatively unexplored area, however the results may not generalize but rather may be transferred to similar groups and settings [38]. Second, the study was not designed as a nested sampling frame, therefore we were unable parcel out differences between public and private schools, schools of varying sizes and grade levels (i.e., elementary and middle schools), and those located in predominately rural, suburban, or urban areas; all characteristics that may influence the results. Because of our focus on potential obesogenic factors, we excluded schools with limited vending and ALC programs. Despite limited competitive food sources, excluded schools had higher obesity rates, likely because they were smaller and poorer. More research is needed to examine potential school-, familial-, and community-level factors contributing to obesity in these resource poor areas. Third, the present study represents the views of high school principals and food service personnel; it does not represent the opinions of teachers, staff, or other administrators (e.g., superintendents, assistant principals).

## Conclusion and implications

This is the first known study to examine the perceptions of high school personnel regarding the school environment, its impact on obesity, and the potential impact of legislation regulating schools' food/beverage offerings. The findings highlight a number of discrepancies between policy makers and school personnel that may inhibit collaborative efforts to address obesity in schools. Our results point to several key factors that may impact policy development and implementation. First, school personnel would like to be included in the development of policies related to the food environment. To date they feel their input and perspective have been largely overlooked. Second, school personnel may not perceive a need for many of the current policy initiatives, which may impact their willingness to comply with policy recommendations. Educating personnel on the link between the school environment, adolescent nutrition, and their ability to make a difference in the childhood obesity epidemic may be a necessary first step in the process. Additionally, framing initiatives in terms of their potential impact on the issues of most concern to schools, particularly improved academic achievement, may increase schools' willingness to initiate environmental changes. Finally, for policies to be successfully implemented they must take into account the barriers faced by schools and minimize burden. Schools cannot implement changes without adequate time and resources. In addition, many schools rely on the revenue from ALC and vending to keep their food service programs functioning and to maintain student participation, and policies that would eliminate or substantially diminish this revenue may not be feasible under current budgetary conditions. Instead, policies must strike a balance between what is best for children and feasible for schools. More research into how schools can provide healthful ALC and vending options without losing valuable revenue is needed.

## Competing interests

The author(s) declare that they have no competing interests.

## Authors' contributions

NLN, CAB, PS, CMK were involved in the conceptualization and design of the study; acquisition, analysis, and interpretation of data, and drafting of the manuscript. EFE and JAS participated in the design of the study and helped to revise the manuscript for important intellectual content. All authors read and approved the final manuscript.
